# Decoding the gut-sleep Nexus: a bibliometric mapping of gut microbiota and sleep disorders

**DOI:** 10.3389/fmicb.2025.1598173

**Published:** 2025-06-02

**Authors:** Dingwen Xu, Zihua Lu, Qin Li, Yuchen Cheng, Zhe Yang

**Affiliations:** ^1^Department of Clinic, School of Medicine, Yangzhou Polytechnic College, Yangzhou, China; ^2^Department of Orthopaedics, Shanxi Provincial People’s Hospital, Taiyuan, China

**Keywords:** gut microbiota, sleep disorders, bibliometric analysis, obstructive sleep apnea, hotspot

## Abstract

**Background:**

An increasing number of studies have focused on the interaction between gut microbiota and sleep disorders. However, there is currently no bibliometric analysis of the literature on gut microbiota and sleep disorders. This study employs bibliometric methods to analyze the current research status and hotspots in the field of gut microbiota and sleep disorders, providing a reference for future research in this area.

**Methods:**

Articles related to gut microbiota and sleep disorders were retrieved from the WOS core database, covering the period from the database’s inception to December 31, 2024. After rigorous screening, VOSviewer and CiteSpace were used to conduct analyses on quantity, collaboration networks, clustering, and citation bursts.

**Results:**

The number of articles on gut microbiota and sleep disorders has increased annually, with a significant surge after 2022. China has the highest number of publications, while the United States has the highest citation count. The institution with the most publications is Shanghai Jiao Tong University, and the institution with the most citations is Deakin University. The top 10 journals by publication volume are all ranked above Q2 in the JCR. The most cited article is “Gut microbiome diversity is associated with sleep physiology in humans” by Smith et al., published in PLOS ONE in 2019. The top 10 most frequent keywords are gut microbiota, sleep, depression, inflammation, chain fatty acids, anxiety, brain, oxidative stress, obesity, and health. The keyword cluster “obstructive sleep apnea” is a focal research direction, while fecal microbiota transplantation is a current research hotspot.

**Conclusion:**

This study reveals the publication trends, collaboration relationships among countries, regions, and authors, and recent research hotspots in the field of gut microbiota and sleep disorders through bibliometric methods, providing an objective data reference for scientific research in this domain.

## 1 Introduction

Sleep, as an indispensable component of human health, plays a crucial role in maintaining circadian rhythms. With the continuous advancement and development of society, the proliferation of electronic devices, coupled with the fast-paced lifestyle and high-intensity stress, has led to increasingly prevalent issues such as sleep disturbances, sleep disorders, and even sleep deprivation (Zheng et al., 2024). Sleep disorders are severe sleep-related diseases caused by disruptions in biological rhythms and imbalances in sleep homeostasis (Cohen et al., 2022). Common sleep disorders include insomnia, sleep apnea syndrome, circadian rhythm sleep disorders, narcolepsy, and rapid eye movement sleep behavior disorder (Latreille, 2019). Sleep disorders not only impair cognitive functions, adversely affecting attention, working memory, decision-making processes, and emotional processing, but also interfere with hormone secretion and metabolism, thereby increasing the risk of chronic diseases such as cardiovascular disease, diabetes, and obesity (Antza et al., 2021).

The gut microbiota, as a complex microbial community within the human body, forms a bidirectional regulatory network with the central nervous system through the “brain-gut-microbiota axis” (BGMA) (Naufel et al., 2023). On one hand, gut microbiota influences brain function through three core pathways: immune regulation (interaction with immune cells), neuroendocrine (regulation of the hypothalamic–pituitary–adrenal (HPA) axis and cortisol secretion), and neural conduction (transmission of metabolic products via the vagus nerve) (Asadi et al., 2022). Neuroactive substances such as D-lactate produced by gut microbiota can directly affect the central nervous system, modulating sleep structure and neural function (Zhang et al., 2024). Conversely, the brain can also regulate the composition and metabolic activity of gut microbiota through the same pathways (Morais et al., 2021). Research has confirmed that gut microbiota not only maintains normal sleep rhythms through neurotransmitters and metabolites, but abnormal sleep patterns can also alter the structure and function of the microbiota via the BGMA, leading to metabolic product imbalances, ultimately affecting neurological, immune, and metabolic functions (Matenchuk et al., 2020). This bidirectional interaction mechanism provides significant evidence for understanding the relationship between sleep disorders and gut microbiota.

Despite the increasing research on gut microbiota and sleep disorders, there is a lack of bibliometric analysis in this field, making it challenging for academia to deconstruct the knowledge evolution trajectory and interdisciplinary collaboration networks through multidimensional data. Therefore, this study employs bibliometric methods to search the relevant literature in the Web of Science Core Collection database and analyze research trends in the fields of gut microbiota and sleep disorders, providing scholars in this domain with insights into the current status and hotspots, and offering research directions for researchers in this field.

## 2 Methods

### 2.1 Literature retrieval and selection strategy

**Figure 1:**
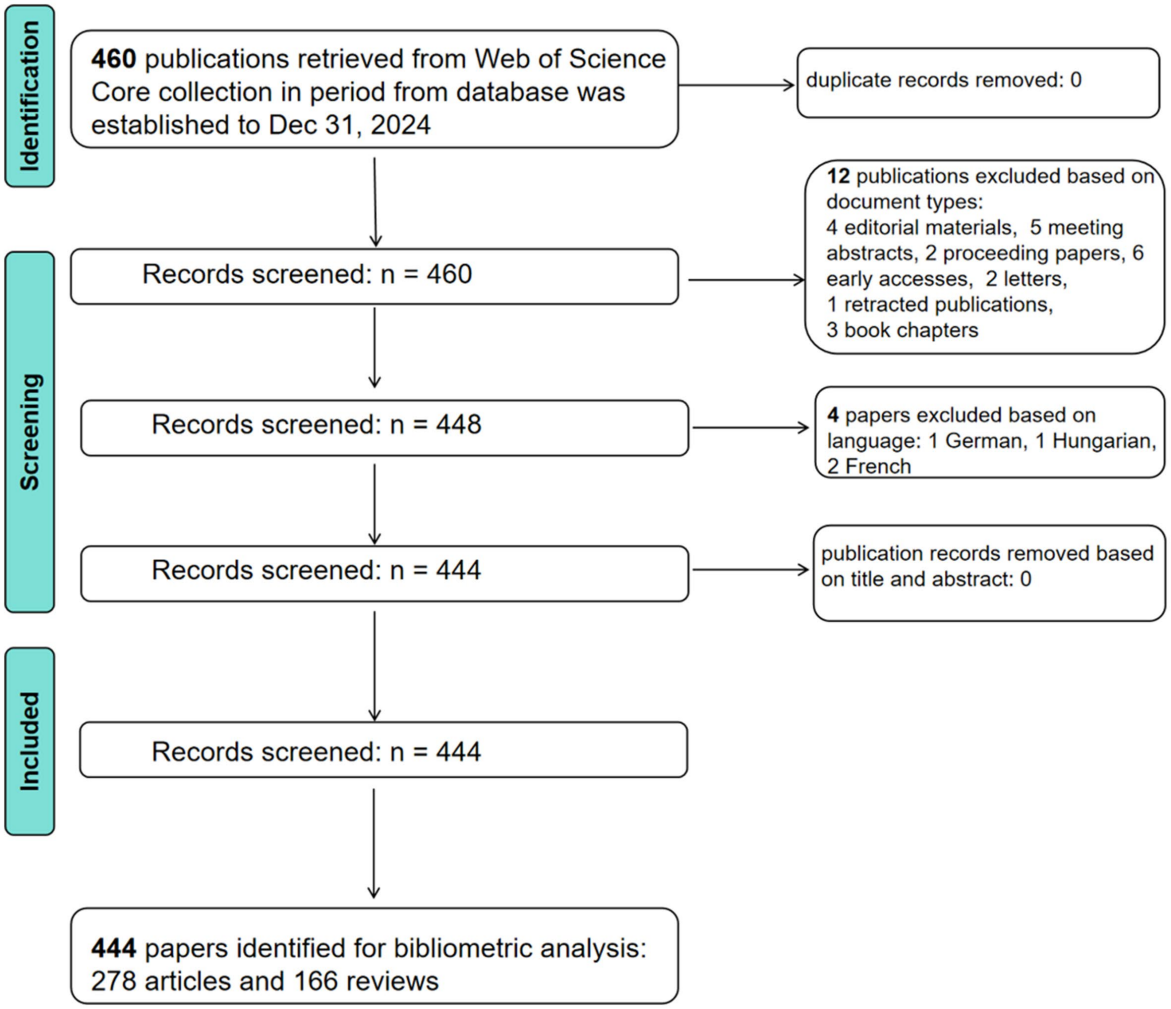
Schematic representation of the literature retrieval and selection.

The Web of Science Core Collection database was queried, with the search period extending from the database’s inception to December 31, 2024. The search terms were not only meticulously crafted using the MeSH (Medical Subject Headings) database to ensure comprehensive retrieval of relevant keywords but also informed by search strategies from prior studies (Dong et al., 2023; Xiu et al., 2023; Shen et al., 2024; Zhu et al., 2024). TS = ((Gastrointestinal Microbiomes) OR (Gastrointestinal Microbiome) OR (Microbiome, Gastrointestinal) OR (GI Microbiome) OR (GI Microfloras) OR (GI Microflora) OR (GI Microbiomes) OR (Enteric Microbiotas) OR (Enteric Microflora Flora) OR (Enteric Microflora Floras) OR (Enteric Microbiota) OR (Gastrointestinal Flora) OR (Gut Flora) OR (Gut Microflora) OR (Gastrointestinal Microflora) OR (Gut Microbiome) OR (Gut Microbiomes) OR (Gastrointestinal Microbiota) OR (Gastrointestinal Microbiotas) OR (Microflora) OR (Microfloras) OR (gut microbiota) OR (gut microbiotas) OR (Gastrointestinal Microbial Community) OR (Gastrointestinal Microbial Communities) OR (Intestinal Microbiome) OR (Intestinal Microbiomes) OR (Intestinal Microflora) OR (Intestinal Flora) OR (Intestinal Microbiota) OR (Intestinal Microbiotas) OR (Enteric Bacteria) OR (Gastric Microbiome) OR (Gastric Microbiomes)) AND TS = ((Dyssomnia) OR (Sleep Disorders) OR (Sleep Disorder) OR (Nocturnal Eating Drinking Syndrome) OR (Nocturnal Eating Drinking Syndromes)). Inclusion criteria: (1) Studies related to gut microbiota and Sleep Disorder; (2) Document types classified as “Article” and “Review”; (3) Language restricted to English. Post-screening, the “Export Records to Plain Text File” option was utilized to export the literature for subsequent bibliometric analysis. Two authors independently conducted literature screening and data extraction. Discrepancies were resolved through discussion with a third author to reach consensus. This review complied with the PRISMA guidelines (Figure 1).

### 2.2 Bibliometric analysis

Bibliometric and visualization analyses were conducted using VOSviewer and CiteSpace software. VOSviewer’s co-authorship function was employed for analyzing collaboration networks among countries, institutions, and authors, while Bibliographic coupling was used for journal coupling network analysis, and the co-occurrence function was applied for keyword co-occurrence analysis. Nodes were included if they met a minimum occurrence threshold of 10 times for keywords or 5 publications for authors or institutions. Select the association strength for the normalization method. The results from VOSviewer can be visualized using density visualization to display the magnitude of documents and citations, overlay visualization to show the timeline of the analysis target, and network visualization to depict the collaboration network of the analysis target. CiteSpace (v6.1. R6) software was utilized for clustering and burst analysis, with “Time Slicing” set from “2010 JAN to 2024 DEC,” “Years Per Slice” set to 1. “Strength” was set to “cosine,” “scope” to “within slice,” and “Selection Criteria” to “g-index.” Burst detection algorithms were employed to analyze citations and keywords, and keyword clustering was performed using a logarithmic likelihood ratio-based clustering algorithm.

## 3 Results

### 3.1 Analysis of annual publications

**Figure 2:**
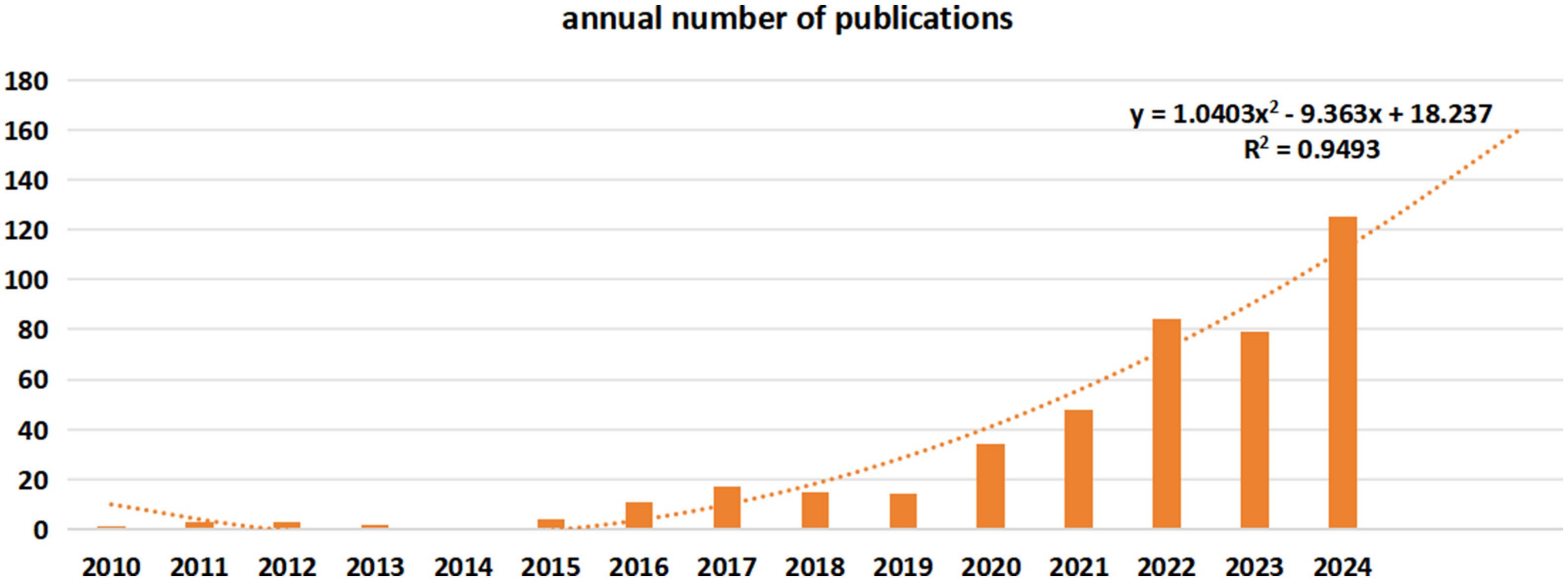
Annual analysis of scholarly publications.

As of December 31, 2024, a total of 444 articles meeting our selection criteria have been published in the Web of Science database, comprising 278 articles and 166 reviews. The inaugural publication concerning gut microbiota and sleep disorders appeared in 2010. As illustrated in Figure 2, the annual number of publications remained below 50 until 2021, with a notable increase to 84 articles in 2022, nearly doubling the count from 2021. In 2024, the number of publications reached 125, representing a 30-fold increase compared to 2014. The polynomial model fitting curve indicates a consistent annual growth in publications related to gut microbiota and sleep disorders, with projections suggesting continued growth (R^2^ = 0.947).

### 3.2 Analysis of countries/regions

**Figure 3:**
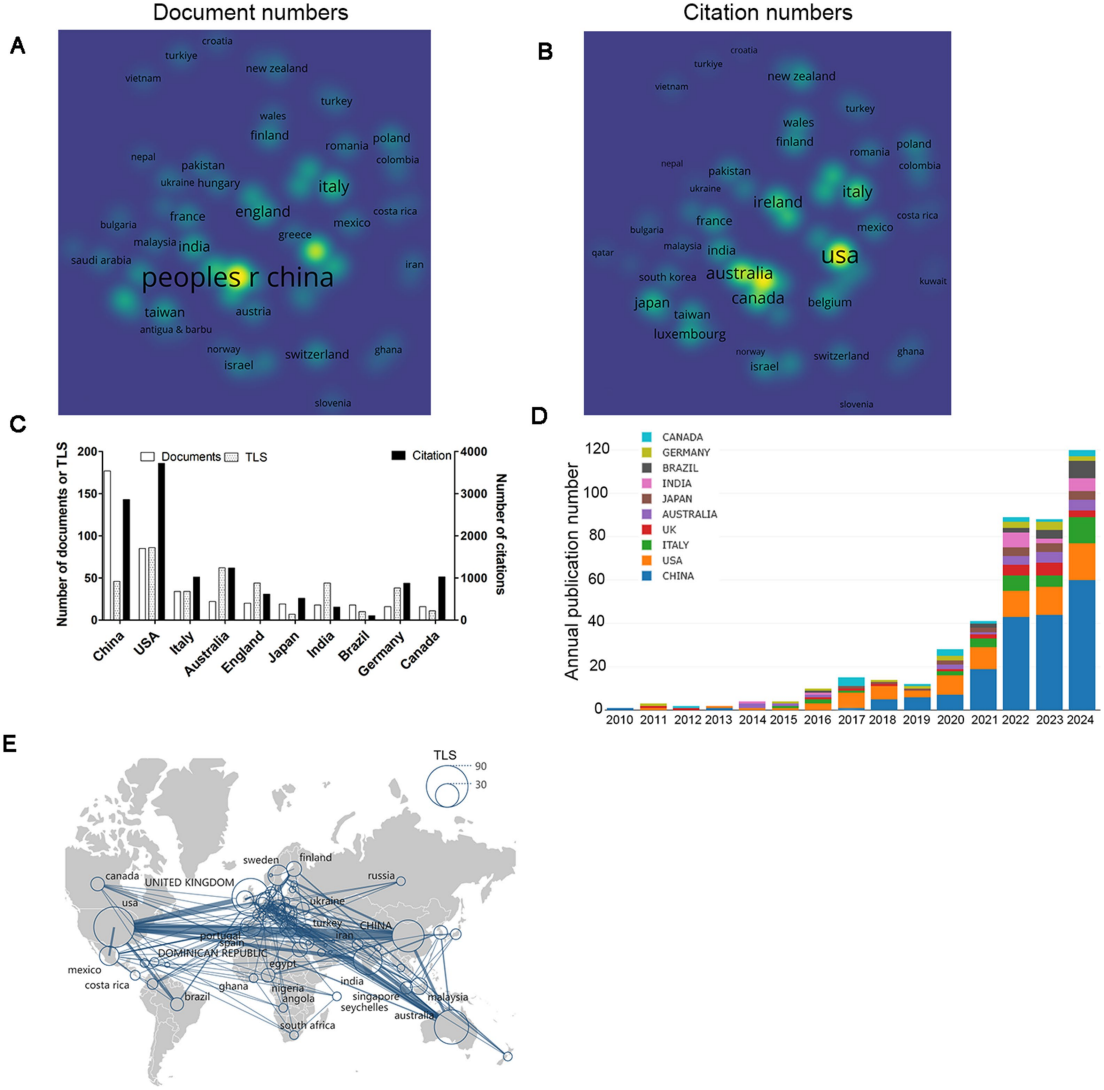
Analysis of countries. **(A)** Density visualization based on the number of documents. **(B)** Density visualization based on citation counts. **(C)** The top 10 countries ranked by publication volume. **(D)** Annual changes in publication volume for the top 10 countries by output. **(E)** Co-authorship analysis of countries/regions depicted on a geographical map. The node size reflects the TLS, and the thickness of the lines indicates the degree of collaboration between the two countries or regions.

A total of 69 countries have contributed to the field concerning gut microbiota and sleep disorders. The publication and citation volumes for each country can be visualized through density visualization maps (Figures 3A,B), where greater brightness indicates higher values. Among the top 10 countries in terms of publication volume, China leads significantly with 177 publications, followed by the United States (*n* = 85) and Italy (*n* = 34). Analyzing the annual publication volumes of these 10 countries reveals that, overall, the United States maintained a leading position in annual publications before 2020, after which China’s annual publication volume began to surpass that of the United States. In terms of total citation counts, the top three countries are the United States (*n* = 3,717), China (*n* = 2,862), and Australia (*n* = 1,235) (Figures 3C,D). A co-authorship analysis of these countries indicates that the United States exhibits the highest level of international collaboration (TLS = 86), followed by Australia (TLS = 62) and China (TLS = 46). Notably, the collaboration between China and the United States is the most robust (Link strength = 13), and there is also significant collaboration between Australia and the United Kingdom (Figures 3C,E).

### 3.3 Analysis of institutions

**Figure 4:**
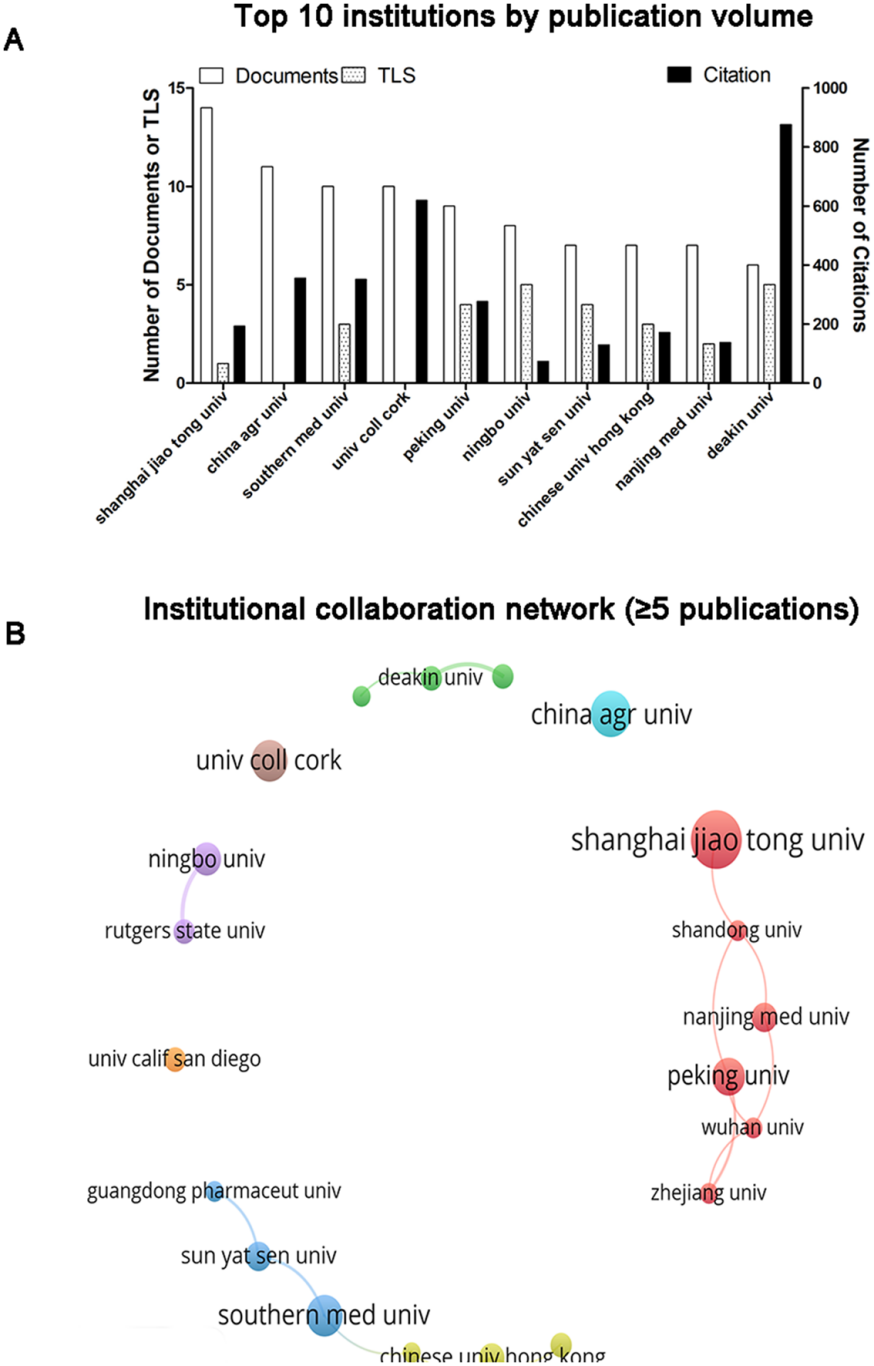
Analysis of institutions. **(A)** The ten leading institutions ranked by publication output. **(B)** Network visualization illustrating institutional collaboration. The node size reflects the TLS, and the thickness of the lines indicates the degree of collaboration between the two institutions.

A total of 915 institutions have published articles on gut microbiota and sleep disorders, with the top 10 institutions by publication volume illustrated in Figure 4A. Shanghai Jiao Tong University leads with 14 publications, followed by China Agricultural University (*n* = 11) and Southern Medical University (*n* = 10). The institutions with the highest citation counts are Deakin University with 876 citations, University College Cork with 619 citations, and China Agricultural University with 356 citations. The strongest collaborative ties are observed among Deakin University (TLS = 5), Ningbo University (TLS = 5), and Peking University (TLS = 4) as depicted in Figure 4A. Additionally, 20 institutions have published more than five articles, and a visualization of the institutional collaboration network reveals that Shanghai Jiao Tong University, Shandong University, Nanjing Medical University, Peking University, Wuhan University, and Zhejiang University form the most collaborative groups. Conversely, institutions such as the University of California, San Diego, University College Cork, and China Agricultural University exhibit no collaborative relationships with other institutions (Figure 4B).

### 3.4 Analysis of authors

**Figure 5:**
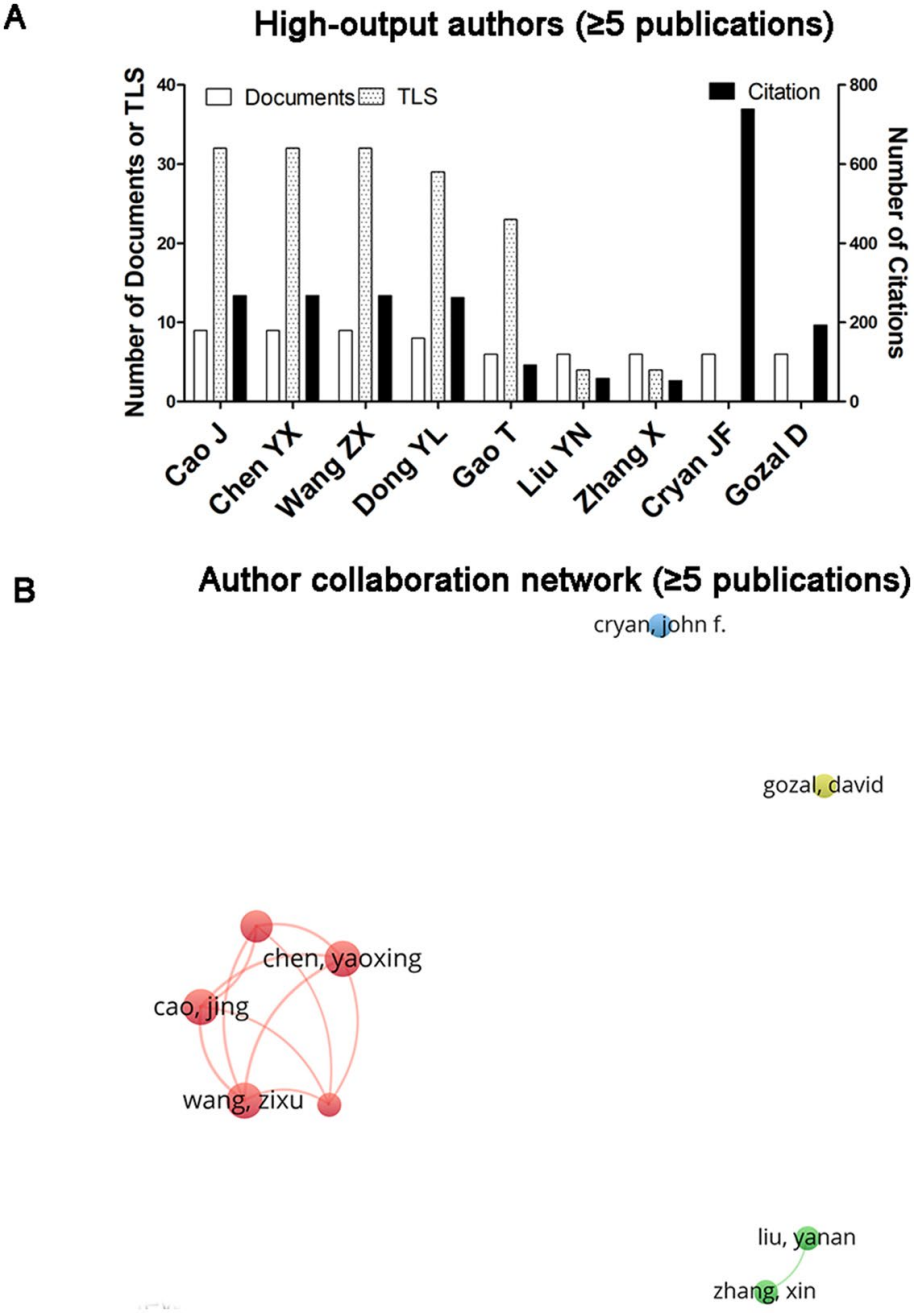
Analysis of authors. **(A)** Publication volume, citation count, and TLS of the nine authors with more than five publications. **(B)** Network visualization of the nine prolific authors. The node size reflects the TLS, and the thickness of the lines indicates the degree of collaboration between the two authors.

A total of 2,870 authors have contributed articles on gut microbiota and sleep disorders. Among them, nine authors have published more than five articles. The publication output, citation count, and degree of collaboration of these nine prolific authors are illustrated in Figure 5A. Figure 5B lists the nine authors with the highest publication volume, highlighting the collaborative relationships among these prolific authors. The cluster formed by Cao J, Chen YX, Wang ZX, Dong YL, and Gao T exhibits the highest degree of collaboration. Cryan JF, although not collaborating with other prolific authors, has the highest citation count (Figures 5A,B).

### 3.5 Analysis of journals

**Figure 6:**
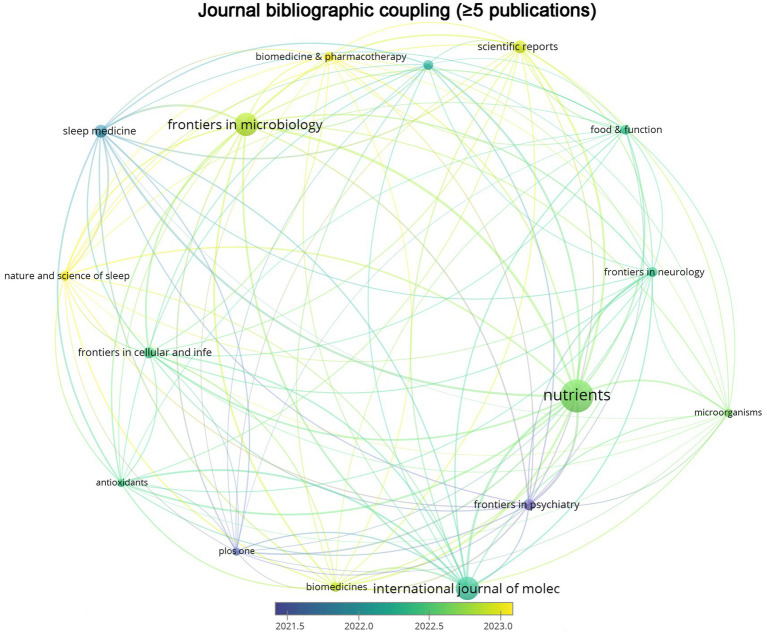
The overlay visualization view of bibliographic coupling across scholarly journals. The size of nodes represents the publication volume of journals, and the thickness of lines indicates the coupling strength between two journals.

A total of 246 journals have contributed articles on gut microbiota and sleep disorders, with 16 journals publishing more than five articles in this domain. The journal “Nutrients” has the highest number of publications (*n* = 20), followed by “Frontiers in Microbiology” (*n* = 14) and “International Journal of Molecular Sciences” (*n* = 14). The citation counts for these three journals are 388, 135, and 464, respectively. The top 10 journals by publication volume are listed in Table 1, all of which have an impact factor greater than 3. Among these, six journals are classified in the Q1 category of the Journal Citation Reports (JCR), while four are in the Q2 category. A bibliographic coupling analysis using VOSviewer on 56 journals with more than five publications revealed that “Nature and Science of Sleep” is a journal with a relatively high recent publication volume, with an average publication year of 2023.00 (Figure 6).

**Table 1:** The ten leading journals ranked by publication output.

Journals	Documents	Citations	Total link strength	Impact factor (2023)	JCR
Nutrients	20	388	1,622	4.8	Q1
Frontiers in microbiology	14	135	1,117	4	Q2
International journal of molecular sciences	14	464	935	4.9	Q1
Sleep medicine	8	161	871	3.8	Q1
Biomedicines	6	62	761	3.9	Q2
Frontiers in psychiatry	7	451	677	3.2	Q2
Antioxidants	5	214	660	6	Q1
Frontiers in cellular and infection mi.	7	90	648	4.6	Q1
Scientific reports	8	77	647	3.8	Q1
Nature and science of sleep	6	109	608	3	Q2

### 3.6 Analysis of references

**Figure 7:**
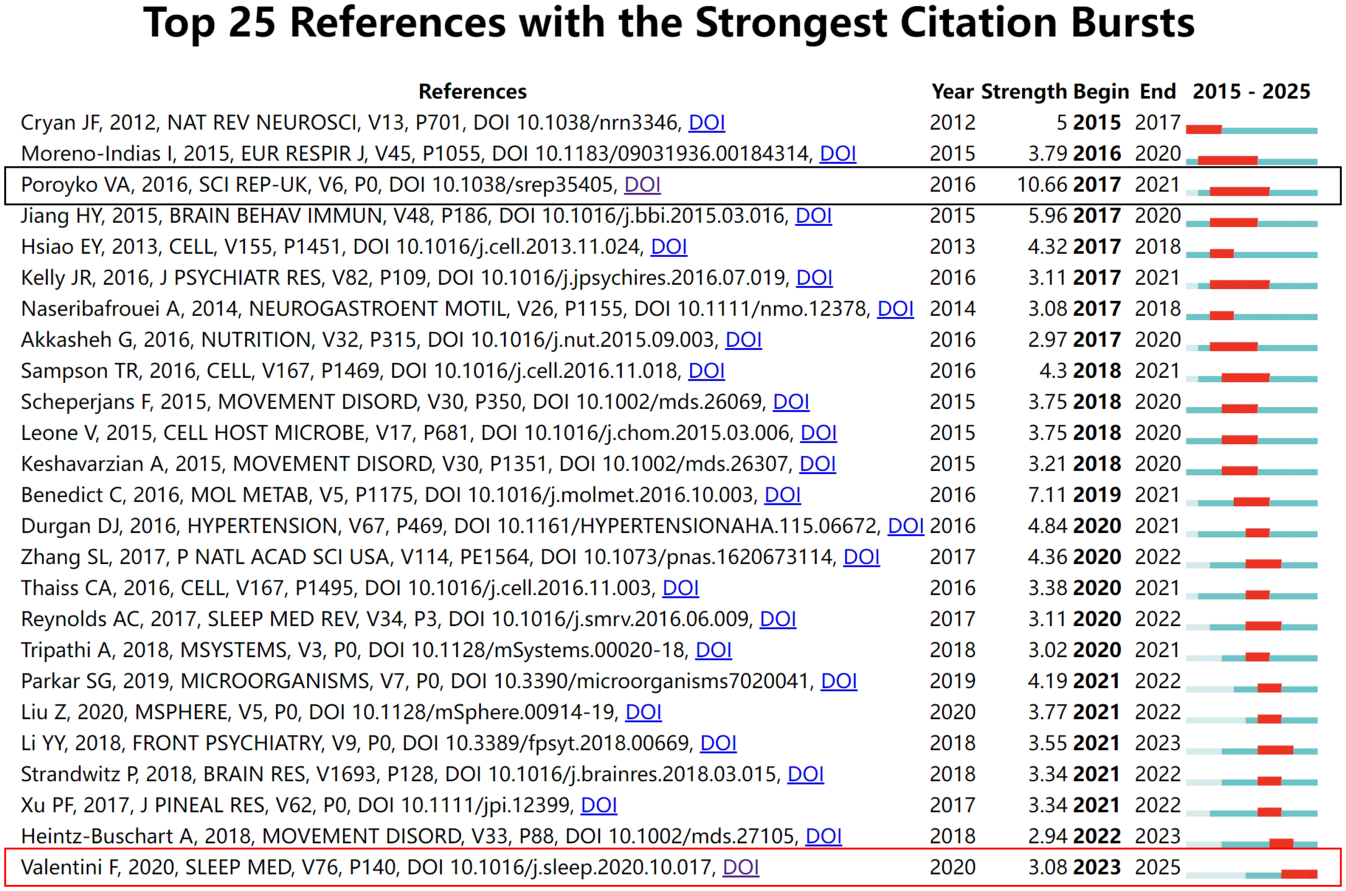
Citation burst analysis of references.

In the research concerning gut microbiota and sleep disorders, a total of 33,342 references have been cited. The most frequently cited article is “Gut microbiome diversity is associated with sleep physiology in humans” authored by Smith et al., published in 2019 in PLOS ONE (Smith et al., 2019), with 41 citations and a citation half-life of 3.5 years. Following this, the article “Sleep, circadian rhythm, and gut microbiota” by Matenchuk et al., published in 2020 in SLEEP MED REV (Matenchuk et al., 2020), has been cited 40 times, with a citation half-life of 2.5 years. Subsequently, the paper “The Microbiota-Gut-Brain Axis” by Cryan et al., published in 2019 in PHYSIOL REV (Cryan et al., 2019), received 37 citations, with a citation half-life of 2.5 years (Table 2). Utilizing CiteSpace for Citation Bursts analysis of references, the top 25 articles with the strongest citation bursts were analyzed. The results revealed that the article “Chronic Sleep Disruption Alters Gut Microbiota, Induces Systemic and Adipose Tissue Inflammation and Insulin Resistance in Mice” by Poroyko et al., published in 2016 in SCI REP (Poroyko et al., 2016), exhibited the strongest citation burst (Strength = 10.66) and the longest duration from 2017 to 2021. Additionally, the study “Gut microbiota composition in children with obstructive sleep apnoea syndrome: a pilot study” by Valentini et al., published in 2020 in SLEEP MED (Valentini et al., 2020), is currently experiencing a citation burst period (2023–2025) (Figure 7).

**Table 2:** The ten most frequently cited references based on citation count.

Title	Journal	First author	Year	Citation frequency	Half-life
Gut microbiome diversity is associated with sleep physiology in humans	PLOS ONE	Smith RP	2019	41	3.5
Sleep, circadian rhythm, and gut microbiota	SLEEP MED REV	Matenchuk BA	2020	40	2.5
The microbiota-gut-brain axis	PHYSIOL REV	Cryan JF	2019	37	2.5
Gut microbiota modulates the inflammatory response and cognitive impairment induced by sleep deprivation	MOL PSYCHIATR	Wang Z	2021	37	1.5
Gut microbiota changes and their relationship with inflammation in patients with acute and chronic insomnia	NAT SCI SLEEP	Li YY	2020	36	2.5
Butyrate, a metabolite of intestinal bacteria, enhances sleep	SCI REP-UK	Szentirmai E	2019	29	3.5
Chronic sleep disruption alters gut microbiota, induces systemic and adipose tissue inflammation and insulin resistance in mice	SCI REP-UK	Poroyko VA	2016	27	3.5
Role of melatonin in sleep deprivation-induced intestinal barrier dysfunction in mice	J PINEAL RES	Gao T	2019	27	3.5
Gut microbiota in obstructive sleep apnea-hypopnea syndrome: disease-related dysbiosis and metabolic comorbidities	CLIN SCI	Ko CY	2019	24	3.5
Reproducible, interactive, scalable and extensible microbiome data science using QIIME 2	NAT BIOTECHNOL	Bolyen E	2019	21	3.5

### 3.7 Analysis of keywords

**Figure 8:**
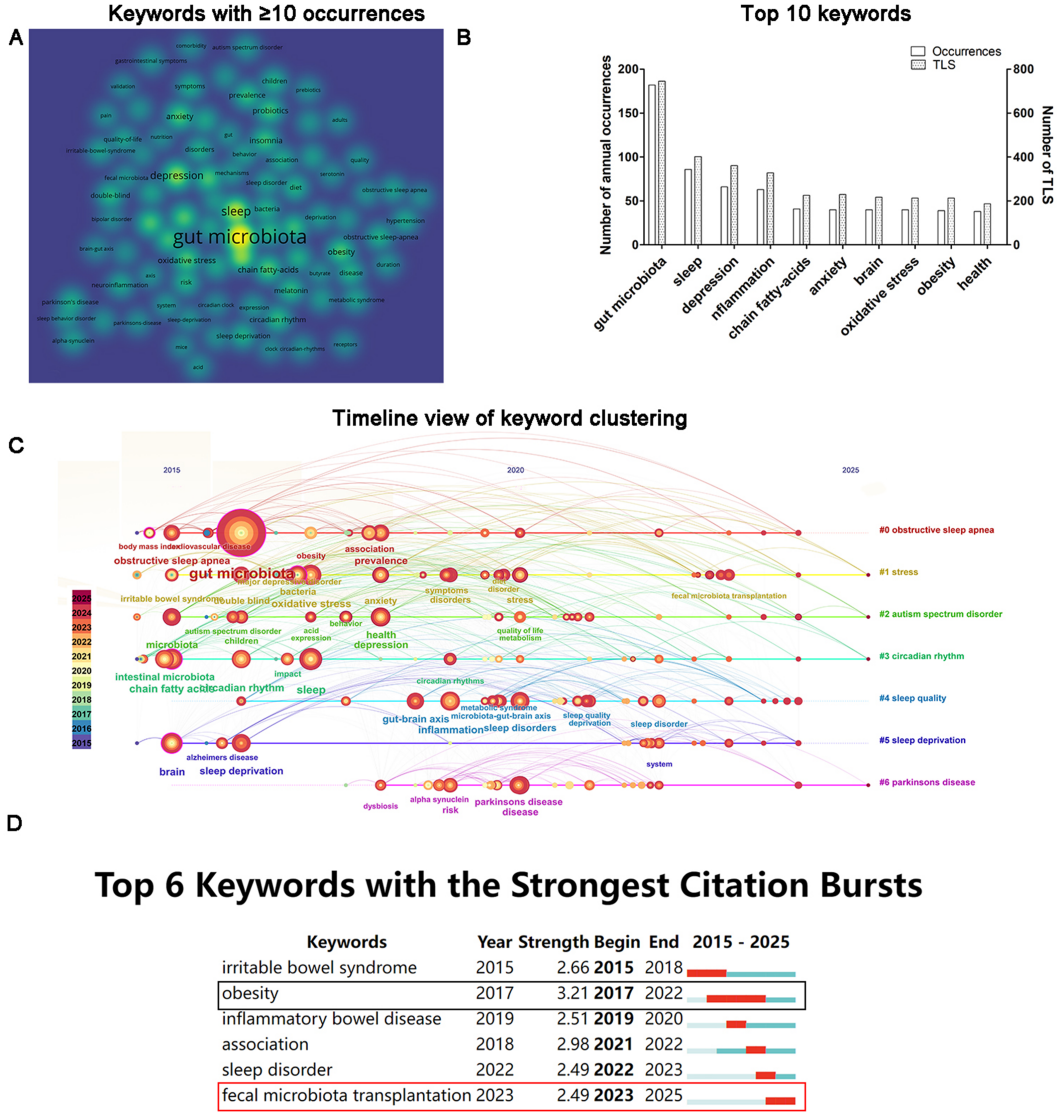
Analysis of keywords. **(A)** The density visualization view of co-occurrence analysis among keywords. **(B)** Top 10 keywords by occurrences. **(C)** A chronological representation of keyword cluster analysis. **(D)** Citation burst analysis of keywords.

Co-occurrence analysis plays a pivotal role in monitoring scientific development by exploring popular research directions and domains through the analysis of relationships between keywords. Utilizing VOSviewer, a co-occurrence analysis was conducted on keywords appearing at least ten times, resulting in the identification of 92 keywords (Figure 8A). The top ten keywords with the highest occurrence are as follows: gut microbiota (occurrences = 182, TLS = 745), sleep (occurrences = 86, TLS = 402), depression (occurrences = 66, TLS = 361), inflammation (occurrences = 63, TLS = 328), chain fatty-acids (occurrences = 41, TLS = 226), anxiety (occurrences = 40, TLS = 1,229), brain (occurrences = 40, TLS = 217), oxidative stress (occurrences = 40, TLS = 213), obesity (occurrences = 39, TLS = 213), and health (occurrences = 38, TLS = 187) (Figures 8A,B). Furthermore, keyword co-occurrence clustering was computed using CiteSpace, revealing that keywords can be categorized into seven clusters: obstructive sleep apnea, stress, autism spectrum disorder, circadian rhythm, sleep quality, sleep deprivation, and Parkinson’s disease (Figure 8C; Table 3). Clusters are numbered starting from 0, with cluster 0 being the largest and cluster 7 the smallest. The “average year” reflects the mean year of keyword occurrence within a cluster, with later years indicating a more cutting-edge research focus. From the perspective of average year of keyword cluster occurrence, sleep quality has the latest average time of occurrence, 2021, highlighting the forefront of research on gut microbiota and sleep disorders. The silhouette values indicate that the closer the value is to 1, the more distinct the theme of the cluster, and the more similar the content of the articles within the cluster, representing a concentrated focus by researchers on the issue (Isola et al., 2025). Obstructive sleep apnea and circadian rhythm have relatively high silhouette coefficients, indicating concentrated research attention in these areas. Keyword citation burst analysis identified six instances of citation bursts, with obesity exhibiting the strongest citation burst (Strength = 3.21) and the longest duration (2017–2022). Additionally, the keyword fecal microbiota transplantation is currently experiencing a citation burst period (2023–2025) (Figure 8D).

**Table 3:** Cluster analysis of keywords.

Cluster ID	Size	Silhouette	Mean (Year)	Label
0	50	0.838	2017	Obstructive sleep apnea
1	44	0.707	2019	Stress
2	44	0.73	2020	Autism spectrum disorder
3	43	0.822	2019	Circadian rhythm
4	38	0.691	2021	Sleep quality
5	35	0.776	2020	Sleep deprivation
6	31	0.749	2020	Parkinson’s disease

## 4 Discussion

Prior to 2021, the volume of publications concerning gut microbiota and sleep disorders was relatively limited; however, there has been a rapid increase post-2022. The marked increase in publications may be attributed to advancements in microbiome research methodologies. Specifically, the reduced costs and enhanced accessibility of 16S rRNA and metagenomic sequencing have facilitated large-scale investigations of the gut microbiome (Wensel et al., 2022). Furthermore, progress in metabolomics and proteomics has spurred mechanistic studies of the gut-brain-sleep interactions (Tilocca et al., 2020). Notably, the top 10 journals in terms of publication volume all possess an impact factor exceeding 3, with six journals classified in the Q1 category and four in the Q2 category according to the Journal Citation Reports. This trend indicates an unprecedented level of interest in this field in recent years. The fitted curve suggests a continued growth in publications related to this domain, underscoring the necessity for a bibliometric analysis at this juncture. Such an analysis would provide researchers, who are fervently engaged in this field, with insights into the current state of research and existing challenges, thereby enhancing the specificity and quality of research outcomes.

China leads in publication volume, yet it is noteworthy that it only surpassed the United States post-2020, reflecting a significant enhancement in China’s research capabilities in this domain over the past five years. This improvement is likely linked to China’s economic development and increased emphasis on scientific research. Furthermore, the highest levels of collaboration, both internationally and domestically, are observed between China and the United States, as well as within these countries’ respective institutions and authors. The observed phenomenon may be attributed to significant research funding from both the United States and China. Specifically, the National Institutes of Health (NIH) and the National Natural Science Foundation of China (NSFC) projects are relevant. Furthermore, the eminence of high-impact institutions in both countries likely fosters increased collaboration due to their established reputations. This collaboration has facilitated the production of research outputs, serving as a model for other nations to strengthen scientific cooperation.

Journal and reference analyses play a guiding role in manuscript preparation and submission. This study identifies the top 10 most-cited articles in the field, indicating their high authority and consensus among researchers. Authors drafting articles in this domain are encouraged to thoroughly review these references and cite them as robust evidence. Additionally, the study reveals that “Nature and Science of Sleep” has published a significant number of articles in recent years, suggesting that submitting to this journal may increase the likelihood of publication success.

Keyword analysis aids in identifying emerging research hotspots. Obstructive sleep apnea (OSA) is a critical factor in sleep disorders with keyword clustering showing the highest silhouette coefficient for obstructive sleep apnea indicating concentrated research interest in this direction (Jordan et al., 2014). Indeed the gut microbiota is implicated in obstructive sleep apnea exerting influence through a multitude of mechanisms. A cohort analysis of 48 OSA subjects revealed a correlation between OSA severity and gut microbiota alterations. The study observed an enrichment of Fusobacterium and Lachnospiraceae_UCG_006 coupled with a reduction in Anaerostipes. Furthermore elevated intestinal barrier biomarkers including D-LA and I-FABP were detected. These findings suggest that gut barrier dysfunction may contribute to systemic inflammation and the development of metabolic comorbidities in OSA patients (Li et al., 2023). Another study using Apoe −/− mice revealed that intermittent hypoxia/hypercapnia (IHC) synergizes with a high-fat diet to exacerbate atherosclerosis mediated by gut microbiota shifts (e.g., Akkermansiaceae enrichment Muribaculaceae depletion) and bile acid dysregulation with aortic lesions being microbiota-dependent while pulmonary artery lesions remain unaffected suggesting microbiota-targeted therapies for OSA-induced vascular damage (Xue et al., 2025). Among the top 10 most frequently occurring keywords the majority are closely related to obstructive sleep apnea such as obesity sleep depression inflammation anxiety and health all of which are intricately linked to the pathogenesis and consequences of obstructive sleep apnea (Vanek et al., 2020; Yang et al., 2023; Messineo et al., 2024). Notably obesity exhibits the strongest and longest-lasting citation burst further highlighting the focus on obstructive sleep apnea within the field. Given the study’s exploration of the relationship between gut microbiota and sleep disorders the keyword “fecal microbiota transplantation” is currently experiencing a citation burst indicating a growing interest in gut microbiota interventions for obstructive sleep apnea with recent emphasis on fecal microbiota transplantation.

Fecal microbiota transplantation has shown considerable promise in treating intestinal infections, inflammatory bowel disease, obesity, diabetes, and hypertension (Rinott et al., 2021; Luqman et al., 2024). In a study involving C57BL/6 J mice exposed to intermittent hypoxia, fecal samples were collected for fecal microbiota transplantation, resulting in increased sleep duration and frequency during the dark cycle in recipient mice (Badran et al., 2020). Additionally, fecal microbiota transplantation has been employed to assess the transferability of obstructive sleep apnea-induced hypertension in animal models, with recipient mice of obstructive sleep apnea donor feces exhibiting significantly elevated systolic blood pressure (Durgan et al., 2016). A Swedish study analyzing respiratory parameters and gut microbiota in 3,570 individuals aged 50 to 64 suggests that hypoxia related to obstructive sleep apnea, rather than the frequency of apneas/hypopneas, is associated with specific gut microbiota species and their functions (Baldanzi et al., 2023). These studies demonstrate the influence of gut microbiota on obstructive sleep apnea and highlight fecal microbiota transplantation as a pivotal research tool, raising intriguing and promising questions about its potential therapeutic application for obstructive sleep apnea.

## 5 Limitations

This study has several methodological constraints that warrant consideration. Firstly, the literature included in our study was restricted to the WOS database and did not incorporate other major databases such as Scopus, PubMed, or Embase. Additionally, articles in languages other than English (e.g., German or French) were excluded, which may have resulted in the omission of potentially significant studies. Secondly, the operational definition of “sleep disorders” adopted in our search strategy encompassed a broad spectrum of conditions including insomnia, sleep apnea syndrome, circadian rhythm sleep disorders, hypersomnia, and REM sleep behavior disorder. The lack of condition-specific analyses may have obscured important pathophysiological distinctions in gut microbiota profiles across different sleep pathology subtypes. Lastly, citation bursts are often influenced by self-citations and journal impact factors, potentially reflecting transient “hot topic” trends rather than sustained scientific impact. Future studies should conduct longitudinal tracking of FMT-related citations to evaluate their long-term validity.

## 6 Conclusion

In summary, the relationship between gut microbiota and sleep disorders is garnering increasing attention in contemporary neurogastroenterology research. Through systematic bibliometric analysis, this investigation elucidates the current research landscape by mapping international collaborations, institutional contributions, and author networks. The identification of high-impact journals, seminal references, and keyword clustering patterns provides valuable insights into evolving research trends. These findings offer strategic guidance for researchers by highlighting knowledge gaps and suggesting methodological refinements. Future studies should broaden database coverage, integrate comprehensive literature, and refine search algorithms using precise MeSH terms and diagnostic criteria to enhance the precision of interdisciplinary bibliometric analyses.

## Data Availability

The original contributions presented in the study are included in the article/supplementary material, further inquiries can be directed to the corresponding author.
